# Measuring generic health using the minimum european health module: does it work and is it better than self-rated health?

**DOI:** 10.1186/s12889-023-16778-2

**Published:** 2023-12-01

**Authors:** Patrick Lazarevič

**Affiliations:** Statistik Austria, Guglgasse 13, 1110 Vienna, Austria

**Keywords:** Health measurement, Minimum european health module, Self-rated health, Generic health

## Abstract

**Background:**

Health is a fundamental aspect of many scientific disciplines and its definition and measurement is the analytical core of many empirical studies. Comprehensive measures of health, however, are typically precluded in survey research due to financial and temporal restrictions. Self-rated health (SRH) as a single indicator of health, on the other hand, exhibits a lack of measurement invariance by age and is biased due to non-health influences. In the three-item Minimum European Health Module (MEHM), SRH is complemented with questions on chronic health conditions and activity limitations, thus providing a compromise between single indicators and comprehensive measures.

**Methods:**

Using data from the German Ageing Survey (2008 & 2014; n = 12,037), I investigated the feasibility to combine the MEHM into a generic health indicator and judged its utility in comparison to SRH as a benchmark. Additionally, I explored the option of an extended version of the MEHM by adding information on multimorbidity and the presence and intensity of chronic pain.

**Results:**

The analyses showed that both versions of the MEHM had a good internal consistency and each represented a single latent variable that can be computed using generalized structural equation modeling. The utility of this approach showed great promise as it significantly reduced age-specific reporting behavior and some non-health biases present in SRH.

**Conclusions:**

Using the MEHM to measure generic (physical) health is a promising approach with a wide array of applications. Further research could extend these analyses to additional age groups, other countries, and establish standardized weights for greater comparability.

**Supplementary Information:**

The online version contains supplementary material available at 10.1186/s12889-023-16778-2.

## Background

Whatever the specific definition, health is a fundamental aspect of people’s lives and, accordingly, of many scientific disciplines. Correspondingly, its definition and measurement are the analytical core of many empirical studies with the validity of results being determined by decisions made in this regard. However, financial and temporal restrictions typically preclude a comprehensive health measurement via extensive scales, performance measures, or the collection of biomarkers. Thus, especially multi-thematic surveys often opt to only ask for the respondent’s self-rated health (SRH) to measure (physical) health as this single indicator potentially provides a comprehensive and inclusive measurement of health and has been shown to predict, among other outcomes, mortality [1, 2].

However, one major drawback of using SRH in empirical research is it’s unclear scaling. As a fully labeled 5 point item, using SRH as a (quasi) metric variable is, to say the least, controversial. Thus, it is typically dichotomized into good vs. poor health [3], resulting in a loss of available variance and restricting its use to certain statistical methods, i.e., methods that are suitable for binary data. To fix this, it would be beneficial to utilize the full variance of SRH in an interval scaled generic health measure to avoid scaling issues and allow for its use in additional statistical procedures.

Further, recent studies have shown that SRH exhibits some properties that question its suitability to validly and robustly measure generic health, e.g., age-specific health determinants and standards, i.e., lack of measurement invariance [4–6], or systematic influences of non-health characteristics even after controlling for comprehensive health information, i.e., non-health biases [7, 8]. Therefore, to efficiently utilize SRH’s potential for inclusive generic health measurement and to increase the validity of research based on SRH, it would be desirable to rectify these drawbacks. As a lot of substantive survey research, e.g., on the relationship of work or family and health, is based on time-sensitive multi-thematic surveys, any attempt to do so has to make use of as little additional questionnaire items as possible.

The Minimum European Health Module (MEHM), as proposed by Robine & Jagger in 2003 [9], complements SRH with a general question on chronic health conditions and another question on any health-related activity limitation, which is also known as the Global Activity Limitation Indicator (GALI). Apart from the obvious brevity of this module and its wide availability as a standard Eurostat module for collecting health information, its indicators other than self-rated health reflect the two most important health domains used by respondents to rate their health as has been shown in more recent research, i.e., functioning and chronic health conditions [5, 6], and cover other degrees of objectivity than SRH.

Thus, the MEHM can, when combined into a single summary score, be seen as a promising compromise between using the highly subjective single-indicator SRH and time-intensive, comprehensive scales. Such a summary measure of generic health could then be used in various ways to measure or account for health. For example, given a sufficient level of comparability, it might be used to compare health outcomes between different societal groups or as a consequence of policy interventions or different health behaviors. Other than that, it might also be used to incorporate overall health as a major independent variable in multivariate analyses, e.g., to study health as a prerequisite for labor market participation or family formation. Lastly, it could also simply be used to ‘control for health’, as is regularly done in multivariate analyses where health is considered to play a significant role but is itself not the focus of the analyses.

The analyses of this paper can be broadly grouped into two parts. Firstly, I investigate the *feasibility *of combining items of the MEHM into a single generic physical health indicator. In order to do so, I compare Cronbach’s $\alpha$ between different age groups to evaluate if the MEHM-items are internally consistent and then use generalized structural equation modeling (GSEM) as a means for confirmatory factor analyses to extract health measures from the MEHM-variables. The remainder of the analyses are focused on judging these health indicators’ *utility* in comparison to SRH as the current de facto state of the art for short generic health measurement by comparing the extent that different non-health aspects bias the health indicators after controlling for a wide array of health information.

## Methods

For the analyses of this paper, I used data from German Ageing Survey (DEAS). The DEAS is a nationally representative panel study for the German population aged 40–85 and consists of four baseline (i.e., cross-sectional) samples from the years 1996, 2002, 2008 & 2014, which were then followed for multiple years in case of giving written consent using questionnaires including topics such as health and well-being. The interviews were conducted using CAPI with an additional drop-off questionnaire left with the respondents to fill out later [10, 11]. I restricted the analyses of this paper to the baseline samples of 2008 & 2014 as the older baseline samples, i.e., 1996 and 2002, lacked various variables needed for the analyses of this paper. Further, I chose not to use any panel waves, to avoid biases due to panel mortality or survivorship biases that can be expected in longitudinal samples. The two data sets used here provided MEHM data for 12,037 respondents (6,102 women and 5,935 men).

As an additional alternative to the standard three-item version of the MEHM, I also explored the use of an extended version of this indicator, tentatively named MEHM+, by adding information on multimorbidity (i.e., 0/1/2+ chronic health conditions) and the presence and intensity of chronic pain. The former addition aimed to give a more detailed view on chronic health conditions as opposed to their broad (non-)existence while I added pain due to its great importance in explaining SRH in previous research [5, 6]. All following analyses in the Results section are shown both for the MEHM and the MEHM+ version separately.

Accordingly, the MEHM(+) variables used in this paper are described in the following. An overview of the distributions of the MEHM(+) variables can be found in Table S1 in the appendix. For a full documentation of all DEAS variables see [12, 13].

- *Self-Rated Health* (SRH): Respondents were asked to rate their present state of health with five answer categories ranging from “*very good*” to “*very bad*”, i.e., the WHO-version of SRH [14]. This variable was used in constructing both MEHM and MEHM+.
- *Chronic Diseases and Health Conditions* (CHRON): As the direct, global question on chronic conditions was first asked in the 2014 wave of DEAS, I reverse engineered this variable using respondents’ reports on whether they were diagnosed with any of 19 chronic diseases and health conditions from a list presented to them, which was first introduced to the survey in the 2008 wave of the survey.^1^ The full list comprised: *high cholesterol; diabetes; high blood pressure; heart attack, angina pectoris; cardiac insufficiency including coronary artery disease; stroke; circulatory disorders in the brain; circulatory disorders in the legs; joint degeneration (arthrosis) of the hips, knees, or spine; osteoporosis; inflammatory joint or spinal disease (arthritis or rheumatoid arthritis); chronic pulmonary disease (e.g., chronic bronchitis, pulmonary emphysema); cancer, malignant tumor (including leukemia); stomach ulcer, intestinal ulcer; incontinence; mental illness (e.g., panic attacks, depression, psychosis); Parkinson’s disease; glaucoma or macular degeneration; other chronic disease or health condition (only longer-term or recurring diseases)*. As CHRON was meant to represent the binary version of chronic diseases and health conditions, I coded this variable as positive if a respondent reported at least one of the conditions on this list. This variable was only used in the MEHM indicator.
- *Multimorbidity* (MULTI): This variable was constructed in a similar way as CHRON with the difference that it distinguishes between 0, 1, and 2+ chronic conditions from the full 19-item list. MULTI was used as part of MEHM+, replacing CHRON.
- *Global Activity Limitation Indicator* (GALI): This variable represents whether the respondents consider themselves to be limited ‘in doing normal activities during the past 6 months due to health problems’ with the answer possibilities (*Yes, limited a lot; Yes, limited a little; No, not limited at all*). GALI was used to estimate both MEHM and MEHM+.
- *Chronic Pain Intensity* (PAIN): To add pain to the MEHM, I used self-reports on whether the respondents viewed themselves as affected by “constant or recurring pain in the last four weeks” and, if they were, the corresponding intensity (*I didn’t have any pain; very slight; slight; moderate; severe; very severe*). This variable was only used to calculate MEHM+ as an additional health indicator.

### The feasibility of combining the MEHM into a single health indicator

As means to quantitatively assess the *feasibility* of combining the MEHM(+) into a single health score, I firstly examined the internal consistency of both versions by calculating Cronbach’s $\alpha$ across three age groups, i.e., 40–54 (n = 3,871), 55–69 (n = 4,408), and 70+ (n = 3,758) to also evaluate if the internal consistency was given across the age spectrum. As the scaling of all items used here can be considered either ordinal or binary, the calculation of $\alpha$ in this paper was based on polychoric correlations [15].

Then, in order to combine MEHM(+) into a single health score, I used Generalized Structural Equation Models (GSEM) with Stata 18.0 [16] to model generic (physical) health as a latent variable underlying responses to the items contained in the MEHM(+). GSEMs are an extension of traditional structural equation modeling, which also allow for the use of ordinal and binary variables and relationships. This method allows for the use of the full variance of SRH and the extracted latent variable is interval scaled, allowing for a broader scope of statistical analyses.

In order to extract the latent variable of MEHM, I ran GSEM-models with a latent variable determining SRH, GALI, and CHRON with the former two being treated as ordinal variables and CHRON treated as a binary variable. The link functions used for this were ordered probit and probit. The latent variable was then estimated for each case in the sample for use in the further analyses. For MEHM+, I did the same with CHRON being swapped out for MULTI and the addition of PAIN as another variable being determined by the latent health variable. In the case of MEHM+, all variables were treated as ordinal by using ordered probit as the link function.

### The utility the MEHM-indicators in comparison to self-rated health

Next, to explore both new indicators’ *utility*, I compared the resulting MEHM(+) measures regarding their susceptibility to age-specific reporting behavior and non-health biases using SRH as a benchmark. To investigate potential age-related biases in reporting one’s health, I Z-standardized SRH and both MEHM(+) scores to enable a comparison on the same scale and then compared the average ‘health’ by indicator over the three age groups mentioned above. Since standards for ‘good’ health appear to be decreasing with age [4, 7], it would be expected that younger respondents’ MEHM(+) scores would be lower than SRH while older respondents’ MEHM(+) scores should reflect a more favorable generic health if these measures are indeed less prone to reporting biases due to age.

As the final part of this paper’s analyses, I analyzed the influence of further non-health biases on each health measure by employing a multi-step analytic approach already described in greater detail elsewhere [8]. In short, the first step of this approach consists of using a linear regression model explaining the generic health measure in question with as many health indicators as available in the data set. For the following analyses, this health model comprised all 19 individual chronic diseases and health conditions as well as the the pain indicator described above, dummy variables for being underweight, overweight, or obese as well as summary measures provided by the DEAS-team regarding the respondents’ physical functioning (a subscale of the SF-36), lung functioning, and depressive symptoms [17] (the full list and the distributions of these variables can be found in Table S2 in the appendix). The full results of these regression models can be found, separately by gender, in Tables S4 & S5 in the appendix of this paper.

In a second step, the residuals from these health data regressions were then used in a second regression model comprising various non-health indicators. To the extent that overall ‘health’ was explained in the first model, any effects in the second model constitute non-health biases. The explained variances due to health information of the three health measures were substantial (Women: $R_{SRH}^{2}$ = .46; $R_{MEHM}^{2}$ = .57; $R_{MEHM+}^{2}$ = .73; Men: $R_{SRH}^{2}$ = .46; $R_{MEHM}^{2}$ = .59; $R_{MEHM+}^{2}$ = .76), pointing to a considerable amount of physical health information being controlled. Consequently, a difference in the effect of a non-health characteristic between two health indicators would suggest a difference in these indicators’ susceptibility to biases due to that characteristic. Because both regression models use data which were partly collected with an additional drop-off questionnaire [17, 18], these analyses were restricted to 7,089 respondents (3,673 women and 3,416 men).

The non-health variables used in these analyses were as follows (the distributions of these variables are shown in Table S3 in the appendix):

- *Education*: According to prior research, respondents with higher formal education appear to rate similar health states more positively [19], possibly due to their ability to use their resources to alleviate negative effects of health problems. However, other research only found this effect for male respondents [8], highlighting the necessity of separate analyses by gender. In the DEAS data, education was available in the form of three groups that are based on the International Standard Classification of Education (ISCED) representing low (ISCED 0–2), medium (ISCED 3–4), and high education (ISCED 5–6) [20].
- *Age*: Age differences in health reporting behavior have long been discussed under such terms as ‘response shift’, referring to a “change in the meaning of one’s self-evaluation of a target construct” [21, 1532] such as health, which are likely due to changing standards for what constitutes, e.g., ‘good’ health [22]. For the analyses in this paper, I used the respondent’s age on the day of the interview as provided by the DEAS-team [17].
- *Region*: With DEAS being a German sample, I found it suitable to incorporate the respondents’ region of residence (Northern and Southern Germany) into this model as it has repeatedly been shown that health (reports) in Germany have a strong regional component with Southern Germans experiencing or reporting better health, e.g., in regards to life expectancy [23, 24] or self-rated health [25], which might be, at least in part, due to differences in living conditions [26, 27]. Accordingly, I grouped the respondents into people living in Southern Germany (Bavaria & Baden-Wuerttemberg) and Northern Germany (all other states) based on the respondents’ residential addresses at the time of the interview [17].
- *Income*: In a similar vein to education, I also incorporated income into the analyses as a more direct measure of resources available to the respondents. In order to use a standardized measure of income between the two survey years, I chose a variable representing the respondent’s income position as the percentage points of the mean equivalent income of the German population in the survey year as provided by the DEAS-team [17].
- *Optimism*: Previous research has shown that optimism mediates the relationship between objective and subjective functioning [28] and that a similar overall health state was more positively rated by respondents with a greater life satisfaction [8]. Thus, I included a measure of optimism in this model to investigate whether potential biasing effects of optimism on SRH can be mitigated using a MEHM-based approach. The measure of optimism used in DEAS measures the expectations of older adults regarding their personal future [29] and was provided as a summary variable ranging from 1 to 4 by the DEAS-team [17].

## Results

### The feasibility of combining the MEHM into a single health indicator

**Fig. 1:**
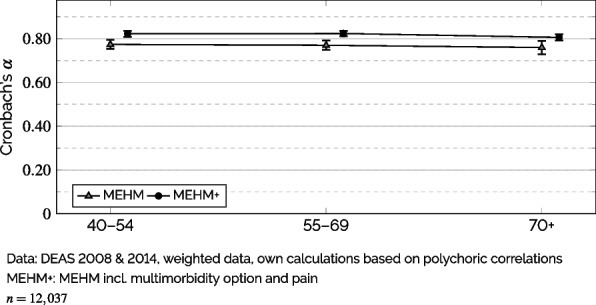
Ordinal $\alpha$ by MEHM-Version and Age Based on Polychoric Correlations (95% CI)

As a first indicator of the *feasibility* of combining the MEHM into a health measure, Fig. 1 displays ordinal Cronbach’s $\alpha$ by age group for both MEHM and MEHM+. These results demonstrate for all three age groups that both MEHM-based health indicators showed a satisfactory internal consistency with $\alpha$ being well above the usual threshold of .70. Moreover, the $\alpha$-values of MEHM+ were significantly greater for all age groups than that of plain MEHM, being consistently above the more restrictive .80-threshold. In short, this suggests both versions of MEHM-based scales exhibited adequate internal consistency for combining them into summary measures of generic health.

**Fig. 2:**
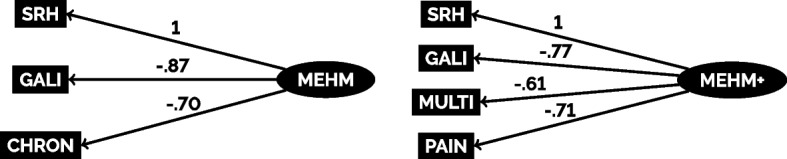
Results from Extracting MEHM(+) Scores as Latent Variables Using Generalized Structural Equation Modeling

The results of extracting MEHM(+) as a latent variable representing generic (physical) health using GSEMs for the two MEHM-variants are presented in Fig. 2. As can be seen here, any effect of both latent variables on any manifest health measures were in line with the expectations as a more favorable rating for SRH indicated a superior latent health whilst greater activity restrictions due to health-problems as well as chronic health conditions lead to a lower generic health score for MEHM. MEHM+ showed the same results with also showing negative effects of multimorbidity and more intense chronic pain on latent health. Accordingly, this points to the estimated health measures being able to be considered to reflect a summary of the health information collected with the MEHM(+) items. All this can be seen as a confirmation of the feasibility of combining the MEHM into a single health indicator by means of GSEM.

### The utility the MEHM-indicators in comparison to self-rated health

**Fig. 3:**
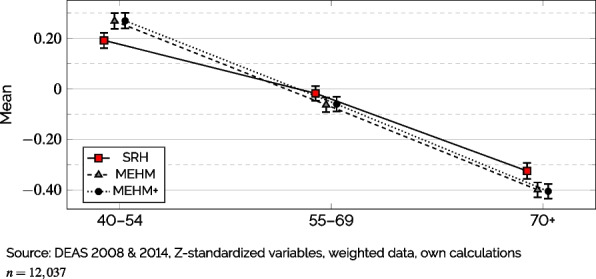
Comparison of Mean ‘Health’ by Age Group and Indicator (95% CI)

In order to judge the *utility* of the previously estimated health indicators based on the MEHM(+), Fig. 3 shows a comparison of the average health scores by the three age groups. For the sake of comparability, I Z-standardized all three health measures, resulting in averages of 0 and standard deviations of 1 for each. Using SRH as a reference, respondents younger than 55 years of age had a significantly better average generic health for both MEHM-measures while respondents above the age of 70 scored significantly lower regarding their generic health. In other words, younger respondents’ overall health scores were, on average, increased for the MEHM(+) scores, while they were lowered for respondents age 70+. As older respondents are typically likely to rate similar health states more optimistically than younger respondents [4, 7], it appears that the MEHM-scores reduced these biases due to age-specific reporting behaviors to some extent. This can be seen as a positive feature of the MEHM(+) measures as this suggests that these health indicators are somewhat less susceptible to age-related reporting biases and, thus, better suited to serve as a measure of generic health across age groups.

**Fig. 4:**
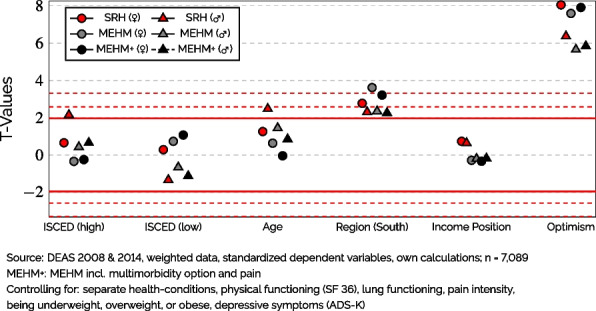
Influence of ‘Non-Health’ Variables on Health Measure After Controlling for ‘Health’ (*T*-values)

Lastly, Fig. 4 displays *T*-values from regressions of non-health indicators on each of the three health measures after controlling for a wide array of health information. The full results of these regressions can be found, separately by gender, in Tables S6 & S7 in the appendix. For ease of interpretation, the most commonly used significance levels of 1.96 (p = .05), 2.58 (p = 0.01), and 3.3 (p = .001) are shown as reference lines. Due to already controlling for health information, any significant effects from non-health variables can be assumed to result either from health characteristics absent from the control model or systematic response biases that are not health-related [8]. Any *T*-values greater than 0 in these analyses indicate that respondents with (a higher value of) that characteristic gave a relatively positive rating to a similar health state based on the health control model. Across all separate models by gender and the three age groups, $R^2$ from health indicators was consistently and markedly lowest for SRH ($\overline{R^2_{adj.}} = .46$) and greatest for MEHM+ ($\overline{R^2_{adj.}} = .74$) with MEHM between the two ($\overline{R^2_{adj.}} = .58$). The detailed results and $R^2$-values by gender for the health control models can be found in Tables S4 & S5 in the appendix.

As can be seen in Fig. 4, a high education had a significant effect, i.e., bias, only for male respondents when using SRH as a measure of health. This indicates that highly educated men with a similar health state, according to the health indicators included in the health model, rated their health more positively than male respondents with a medium education. In contrast, *T*-values for the health indicators based on MEHM(+) were closer to the null line and below the threshold of 1.96 for statistical significance. For women, a high education did not have a significantly biasing effect on any of the three health indicators, which was also true for both genders regarding lower education. However, it can be seen in the figure that the *T*-values of high education were also slightly smaller in the case of women as they were close to zero for both MEHM(+) measures.

Regarding age, Fig. 4 demonstrates a similar picture to higher education as only men exhibited a significant age bias in SRH while the same could not be said about any MEHM score or women in general. However, in the case of age there appeared to also be a consistent gradient with more information going into the indicator resulting in smaller *T*-values. However, due to rather small differences in the overall size of these *T*-values, this finding should not be over-interpreted.

In the case of the region, results were less clear. Regional biases for men were basically unaffected by using the MEHM(+) measures instead of SRH, which were significant on the 5%-level regardless of the health measure used. For women, however, they appeared to be very slightly higher for the MEHM(+) indicators. In the case of MEHM, the *T*-value for being from Southern Germany was significant at the .1%-level instead of the 1%-level, which was the case for SRH and MEHM(+). However, this slight difference should not be over-interpreted as all *T*-values for the regional variable were positive and significant, meaning that Southern Germans rated similar health states more positively than Germans living in the North.

Lastly, in the case of income and optimism it can be noted that the MEHM(+) indicators again seemed to reduce bias in both cases with *T*-values closer to 0, although there were no changes in statistical significance in either case. This means that more optimistic respondents gave a more positive health rating to comparable health states, according to the health model, but this effect was slightly less pronounced in the case of the MEHM(+) measures.

## Discussion

This paper set out to evaluate whether creating a (physical) health score based on the MEHM for older respondents would be feasible using GSEM and whether this indicator would be advantageous in comparison to SRH, as the current standard single-indicator of generic health in survey research, due to potentially being less affected by biases and using all of the collected variation in a metric health indicator. The resulting measure might be used for a wide variety of applications, basically whenever an overall measure of the health status of respondents, i.e., not focused on a specific domain of health, is deemed appropriate for the research question. This explicitly includes research where overall health is merely to be controlled for, studies where health is an important independent variable, or analyses in which general health is the focused outcome, e.g., in a (quasi-)experimental setting.

As the MEHM is a standard module of Eurostat for collecting health information, it is regularly available at least in European surveys that are not limited to a single question on health. For researchers or research groups designing their own surveys, it also gives a widely available and comparable standard to collect health data. Even in cases where this questionnaire module is not directly available in its standardized form, given that the items comprising the MEHM reflect rather common health information, the measure presented in this paper can oftentimes easily be constructed or approximated from health measures available in many surveys. Thus, using the proposed approach, or a variation of it, to measure generic (physical) health is feasible in a wide array of multi-thematic surveys.

In terms of *feasibility*, the analyses have shown that both the original MEHM as well as an extended version (MEHM+) exhibited sufficient internal consistency across all analyzed age groups according to Cronbach’s $\alpha$ based on polychoric correlations due to the variables’ binary and ordinal scaling. Estimating MEHM(+) scores was possible by extracting (physical) health as a latent variable using Stata’s GSEM and showed the expected results regarding the signs of each relationship between indicators and the latent variable.

As for the *utility* of these new health measures, further analyses on potential non-health biases have shown that the two MEHM(+) indicators reduced biases due to age-related reporting behaviors and did not exhibit the statistically significant biases due to high education in men as SRH did. Further, a reduction in bias due to high education for women as well as income and optimism for both genders was apparent, although to a lesser extent. Contrarily, region biases were basically unaffected by using the new measures.

Of course, this paper can only give a first introduction to these measures and future research in additional aspects of this measure would be desirable to give a more detailed picture of the utility of the MEHM(+) approach. One such issue would be more detailed research into regional comparability of these measures. As other research has already shown, SRH can be biased due to the country of residence of the respondents [8, 30], threatening its cross-country comparability. And even on a lower level or regional aggregation, SRH can suffer from biases due to regional response biases, as has also been shown, for example, in this paper. Therefore, it would be interesting to see if the MEHM(+) approach might be less susceptible to reporting behaviors of this kind and whether it would be possible to further attenuate these biases in the MEHM(+) framework. Similarly to regional biases, SRH has been shown to suffer from an insensitivity to health changes [22, 31] with changes in SRH also appearing to be based on changes in different health domains depending on the cohort of the respondent [6]. Future research could investigate whether the proposed health measures are better suited to be used for measuring changes in health or if they can improve upon SRH’s well-known prognostic power regarding mortality [1, 2].

Despite the promising first results on these novel health measures presented in this paper, both the data used here as well as the GSEM-approach in general have some important limitations. Firstly, the DEAS is restricted to only older respondents in Germany and, thus, cannot be generalized beyond this scope. Further research on MEHM-based indicators with different samples would be highly desirable, especially to see the utility of this approach to standardize the measurement of health across age groups, which might be considered a major issue for SRH [5, 6].

As with all secondary analysis, of course, the DEAS was also limited in the available (non-)health data that might be relevant for this research. For example, it might be interesting to see whether potential biases due to hypochondriasis can be reduced in MEHM(+), as they might be major determinants of SRH [32].

Secondly, the approach of using GSEM to compute MEHM(+) scores as a whole has some limitations it shares with any latent variable or factor analysis approach. For example, any indicators resulting from such analyses are standardized to the specific data they are based on. Thus, while they might be useful to operationalize generic health in a specific study, they cannot be compared between two separate data sets, which might be desirable for some research questions. However, this issue might be alleviated by establishing ‘standard weights’ from large general population samples in the future and using these to estimate comparable MEHM(+) scores across data sets.

Another potential drawback of the approach is that the interval scaling of the MEHM(+) scores does not offer any obvious threshold for dichotomization. While this is not a problem for using the MEHM to measure generic health to include it, for example, in regression analyses, a distinction between ‘good’ and ‘bad’ health is necessary for some statistical procedures, such as the calculation of health expectancies [33] or survival analyses. In order to use the MEHM to carry out analyses that necessitate binary health outcomes, other approaches to combining MEHM(+) data might be considered, e.g., latent class analysis. Further research on this use of the MEHM would be desirable.

Lastly, given GSEMs not being a standard method for some researchers, additional computational efforts might hinder the use of MEHM(+) scores in applied research. If the approach can be shown to be useful in future research and more established, however, this issue might be solved by data suppliers simply providing such measures along with their data sets, especially if the standardized weights mentioned above are already available.

## Conclusions

This paper has shown that an indicator based on the MEHM is a feasible and potentially useful, robust alternative to SRH as a generic (physical) health measure. Other than SRH, this health score avoids the typical scaling issues by virtue of being interval scaled and uses all information contained in the variable unlike typical dichotomization approaches for SRH. By using two additional questionnaire items, the described approach offers a method to measure generic health in a brief way that is more inclusive, robust, and exhibits less non-health biases than SRH alone. As such, this instrument might help to increase the validity of health-related empirical research. Further, it can be used in many multi-thematic surveys given that it is based on an already established Eurostat questionnaire module, which can easily be reverse engineered in many other surveys due to the general nature of its content. The approach is also flexible in extending the health score with additional answer categories or other items, if available, to further improve the measurement as done in this paper with multimorbidity and pain. However, further research is needed to test its utility in more contexts and with other data sets.

### Supplementary Information

**Additional file 1: Table S1.** Descriptive Statistics on Gender, Wave, and the MEHM(+) Variables by Year. **Table S2.** Descriptive Statistics on the Health Variables by Year. **Table S3.** Descriptive Statistics on Gender, Wave, and the Non-Health Variables by Year. **Table S4.** Regression Results for the Health Model (Female Respondents) by Generic Health Measure. **Table S5.** Regression Results for the Health Model (Male Respondents) by Generic Health Measure. **Table S6.** Regression Results for the Non-Health Model (Female Respondents) by Generic Health Measure. **Table S7.** Regression Results for the Non-Health Model (Male Respondents) by Generic Health Measure.

**Additional file 2.** Stata syntax used to extract the MEHM(+) scores from the data.

## Data Availability

The data are available for scientific research from the German Centre of Gerontology (https://www.dza.de/en/research/deas).

## Abbreviations

SRH: Self-rated health
MEHM: Minimum European Health Module

## Footnotes

1 An analysis based on 2014’s single question instead of this version, which was consequently restricted to the 2014-sample, both for CHRON and MULTI lead to generally the same results.
